# Overcoming kidney organoid challenges for regenerative medicine

**DOI:** 10.1038/s41536-020-0093-4

**Published:** 2020-04-30

**Authors:** Thomas Geuens, Clemens A. van Blitterswijk, Vanessa L. S. LaPointe

**Affiliations:** MERLN Institute for Technology-Inspired Regenerative Medicine, Maastricht University, Maastricht, The Netherlands

**Keywords:** Translational research, Stem-cell research

## Abstract

Kidney organoids derived from human induced pluripotent stem cells bear the potential to be used as a regenerative medicine renal replacement therapy. Advances in developmental biology shed light on the complex cellular regulation during kidney morphogenesis in animal models resulting in insights that were incorporated in the development of groundbreaking protocols for the directed differentiation of human pluripotent stem cells to kidney endpoints. Moreover, further optimization efforts to improve three-dimensional culture techniques resulted in the creation of kidney organoids. Before they can find their way to the clinic, there are critical challenges to overcome. Here, we will discuss recent advances and remaining challenges for kidney organoids to become successful in regenerative medicine. An innovative combination of tissue engineering techniques with more refined insights in the developing human kidney will ultimately lead to more mature and functional kidney organoids suitable as renal replacement therapy for patients with chronic kidney disease.

## hPSC-derived kidney organoids for regenerative medicine

**Fig. 1:**
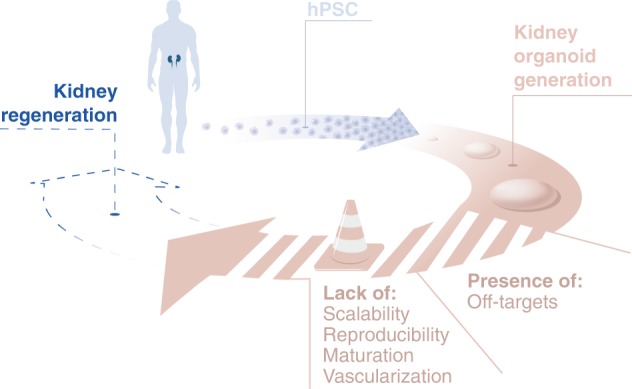
Renal replacement therapy in regenerative medicine. Stem cell-derived kidney organoids are a promising regenerative medicine therapy for patients with chronic kidney disease. Before it can be applied to the clinic, crucial improvements on off-target populations, maturation, vascularization, reproducibility and scaling are required.

Chronic kidney disease (CKD) currently affects 11–13% of the global population and its prevalence is increasing^1^. The high prevalence of CKD in the elderly is the result of a combination of age and cardiovascular risk factors^2^, while the most common causes in youngsters are pediatric tumors (e.g., Wilms tumor) and congenital anomalies of the kidneys and urinary tract (CAKUT)^3^. As a result, two million people worldwide are dependent on dialysis with no option for renal transplantation^4^. Given the high mortality rate and the socioeconomic burden of dialysis, there is a crucial need for alternatives. The regenerative medicine field aims to find these alternatives in the form of kidney organoids derived from human pluripotent stem cells (hPSCs)^5–7^. Generating hPSCs from healthy donors or patients with kidney disease also enables the creation of immunocompatible and patient-specific solutions (Fig. 1)^8^. It is obvious that the generation of hPSC-derived kidney organoids holds great promise as an innovative source of functional nephrons for patients with CKD, but there are crucial challenges to first overcome.

## Advances in kidney organoid generation

**Fig. 2:**
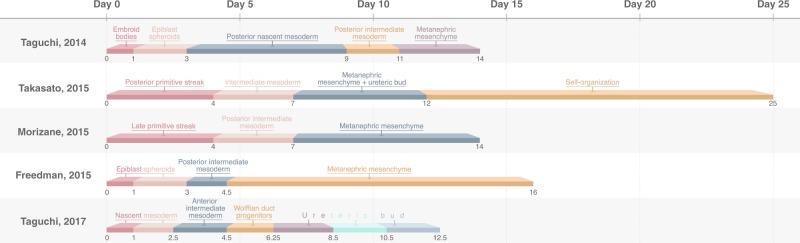
Kidney organoid differentiation protocols. Schematic overview of all established kidney organoid protocols starting from hPSCs. The timeline shown on top indicates the number of days the protocol takes to induce the developmental stages in vitro. Color-coded boxes are not unique, but help visualize the different key steps in the respective protocols. Note that the protocol described here by Taguchi, 2017 highlights the differentiation process to ureteric bud cells and is an addition to their previous protocol (Taguchi, 2014) that exclusively induces renal progenitor cells.

Inspired by developmental biology, numerous protocols for generating organoids starting from pluripotent stem cells have been established (Fig. 2). In the developing embryo, hPSCs only exist transiently and rapidly differentiate into the many specialized cell types that make up the human body^9^. The discovery of a defined set of transcription factors to reprogram terminally differentiated cells resulted in a more flexible access to pluripotent stem cells without the need for human embryos^10^. Interestingly, stimulating their intrinsic properties to differentiate into other cell types by changing the composition of growth factors and culture conditions led to the formation of derivatives of the three primary germ layers: endoderm, ectoderm, and mesoderm. Deciphering the required biochemical and biophysical cues resulted in protocols that were able to generate different cell lineages, including pancreatic, cardiac and neuronal fates^11^. Unfortunately, these protocols often led to the generation of immature, embryonic cultures in terms of differentiation and functional capabilities compared to their adult counterparts.

Finding an efficient and reliable differentiation protocol for renal cell types from mouse embryonic stem cells (mESCs) and hPSCs is a more recent outcome dating from 2013^6,12–14^. Shortly after, additional, but separate, studies by Takasato et al. and Morizane et al. reported differentiation protocols that yielded what were termed kidney organoids^5,7^. These protocols are based on the induction of the major steps of renal development with small molecules and growth factors and researchers continue to optimize them as they seek the ideal strategy to create kidney organoids that most closely resemble the human kidney^5,7^. This is no easy feat; the anatomical complexity of this particular organ and its numerous different cell populations are most probably the main reasons why the most substantial advances only occurred in the past 5 years.

The breakthrough information needed for the generation of kidney organoids was a clear understanding of how the kidney develops in vivo and which guiding molecules play an important role. One of the initial reports shedding light on this employed cell lineage-tracing in mice and showed that the generation of all kidney cell populations was dependent on two main progenitor lines^15^. More specifically, the nephrons, the blood filtration units of the kidney, are derived from the metanephric mesenchyme (MM) that arises from the posterior intermediate mesoderm. The second progenitor line, the ureteric bud (UB), arises from the anterior intermediate mesoderm. Both progenitors originate from the posterior primitive streak formed in the blastocyst.

Using this knowledge from development, Taguchi et al. developed a protocol to induce the MM from both mESCs and hPSCs by optimizing the spatiotemporal application of a defined set of guiding molecules. Although this report of directed renal differentiation was promising, differentiating the hPSC aggregates, so-called embryoid bodies, resulted in a heterogeneous and uncontrollable outcome. An hPSC monolayer was used instead and the optimization of key differentiation steps led to an improved protocol able to simultaneously generate the two main renal progenitor lines (MM and UB)^5^. In order to allow for the formation of more complex multicellular structures that have an improved anatomical representation, differentiated hPSCs were aggregated and grown at the air-liquid interface on a transwell filter^5^. Remarkably, these organoids also developed a collecting duct epithelium together with interstitial and endothelial cells. Although the presence of these new residents was appreciated, they were still in the minority compared to other renal cell types. Together with expression profile studies, these kidney organoids have been found to be developmentally immature as their transcriptome most closely reflects human fetal kidneys in the first trimester of gestation^5^. Interestingly, introducing them to a microenvironment in combination with defined inductive cues resulted in the generation of more mature organoids resembling second trimester fetal kidneys^16^. Although these organoids were generated using a different differentiation protocol, it highlights the importance of the right biochemical and biophysical cues to control cell differentiation.

A third publication, published at the same time, came from Morizane and colleagues. Their protocol differs from Takasato’s because only the posterior intermediate mesoderm was induced to obtain nephron progenitors^7^. Not inducing the anterior intermediate mesoderm resulted in the absence of a collecting duct epithelium as well as interstitial and endothelial cells^17^. In a follow-up study, a new protocol was proposed to generate nephron structures starting from hPSCs embedded as spheroids in Matrigel^18^. These spheroid cultures made the transition towards tubular organoids containing nephrons but also share markers for proximal tubules, podocytes, and endothelial cells.

More recently, Taguchi et al. updated their protocol to generate both UB and MM. Because these progenitors originate from distinct regions in the IM (anterior and posterior) the authors generated each separately which were then combined by the aggregation to allow for self-organization. In order to mimic higher-order kidney organoids, the co-culture was supplemented with renal stroma from mice, resulting in branched ureteric trees enclosed with nephrons^19^. Although these results are extremely promising, the final obtained structure is unstable in long-term culture. The lack of human renal stroma to provide biophysical and biochemical cues could be a plausible explanation.

Recently obtained knowledge of renal development during embryogenesis laid down the base for the generation of currently used protocols. Although the generation of the human kidney is a rather complex event, kidney organoids are typically differentiated from hPSCs by stimulating only a limited set of signaling pathways. To increase cellular complexity and three dimensionality, organoids are dependent on their intrinsic self-organization properties (see reviews refs. ^20,21^). The challenge in this approach is that this much-needed self-organization turns out to be very hard to control.

## Challenges for kidney organoids in regenerative medicine

**Fig. 3:**
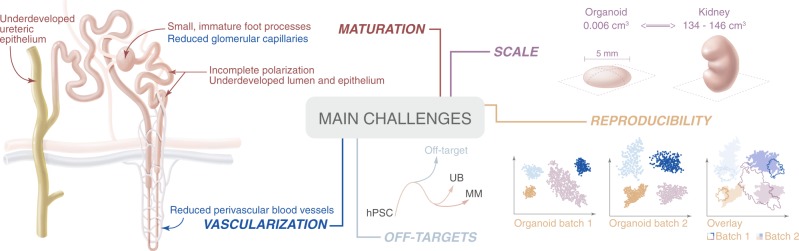
Kidney organoid challenges faced today in the field of regenerative medicine. There is a current lack of full maturation, well-developed vascularization and a limitation in scalability and reproducibility. In addition, there is the presence of non-renal off-target populations. These limitations need to be addressed before kidney organoids can be successfully applied as a replacement therapy for patients with kidney disease.

With impressive progress being made to direct the differentiation of hPSCs to kidney endpoints, a replacement therapy for regenerative medicine may become reality. This will fill the urgent need for alternative treatment as currently dialysis is associated with high morbidity and renal transplantation faces donor transplant shortages. But before kidney organoids will be suited for replacement therapy, there are challenges that should first be addressed (Fig. 3). With a combination of our increasing knowledge in cell biology and the introduction of technologies from the tissue engineering discipline, we propose that these challenges can be overcome.

### Reducing off-target populations

Single-cell transcriptomics of kidney organoids derived from two established protocols identified up to 20% of the cell population to be non-renal^22,23^. These off-target cells include neuronal, muscle and melanoma cell populations that increase in prevalence when organoid cultures get older and take over from the renal cell populations. Earlier studies also reported the presence of fibrotic regions and the appearance of cartilage^7,24^. The generation of these off-target populations could be explained by the way these protocols direct uncommitted mesodermal cells towards specific cell fates. By applying only one directional cue, which is the mesodermal patterning to primitive streak, a variety of mesodermal endpoints is very likely^25^. The presence of these different endpoints has been appreciated in the amount and distribution of distinct cell types in kidney organoids when generated using different protocols. Understanding the lineage relationships and the interconnectivity of signaling networks during differentiation is required to make significant improvements to current protocols. Analyzing transcription factors and signaling networks before and after each branch point will enable us to better control organoid cell fate. Besides understanding the biochemical cues, it is also crucial to better understand cell-extracellular matrix (ECM) interactions to further build up spatial organization. It is clear there is a need to control for self-organization in order to improve the specificity of the renal cell populations in current kidney organoid cultures.

Although a major driver of embryogenesis, self-organization turns out to be challenging to implement for the organoid field^26^. Incorporating some of the innovative tools that are nowadays available to provide cells with specific steering cues to drive their organization could improve this. For example, cell fate and organoid formation can be guided by using synthetic hydrogels that are able to modulate key parameters^27^. These signals are two-fold, being both biochemical and biophysical. Incorporating a hydrogel with guidance molecules based on cell biology knowledge, or by varying its stiffness or degradability could provide a more directed control of organoid self-organization^27^. For example, the use of soft hydrogels to differentiate hPSCs into renal progenitors has proved to be a significant improvement to the protocol^16^. Further fine-tuning the organoid microenvironment by applying physical cues resulted in the formation of larger tubules^28^. In addition, synthetic hydrogels bare the ability to controllably release therapeutic agents such as small molecules and macromolecular drugs^29^. By forming a physical interaction with the polymer, the timing and location of the release can be determined by tuning its physical properties, such as bio-degradability, or by hydrogels that respond to cellular cues^29^. By obtaining more insights in the physical and chemical properties of materials and by achieving a better understanding of the etiology of off-target populations, the field is expected to benefit from the use of biomaterials for controlling cell fate in the following years.

### Promoting maturation and vascularization

With the current protocols for generating kidney organoids in vitro based on mimicking human embryogenesis, it is no wonder that the resulting structures are models of the developing kidney but not of the mature organ. For example, no kidney organoid to date shows an established ureteric epithelium or a patent vasculature. For regenerative medicine, obtaining a mature organoid will be crucial to achieve vascularization and to minimize the chances of unwanted differentiation processes after transplantation and for the function of the tissue.

Global expression profiling of organoids revealed the highest similarity with first and second trimester human kidneys^5,16^. Additional support for this finding came from a recent work that showed transcriptional overlap between organoids and human fetal kidney for key nephron, endothelial, and stromal cell markers^22^. Interestingly, the transcriptional variability appreciated between experimental batches was particularly observed in genes typically expressed in older organoids^30^. This could indicate that the lack of maturation lies in differences in gene expression profiles of distinct cell populations.

Functional kidneys are dependent on a regulated supply, and disposal, of biomolecules and need a continuous filtration of blood to maintain body fluid homeostasis. This relies on a highly developed vasculature network surrounding the tubular compartments and perfusing the glomeruli with blood capillaries. Immunohistochemistry and gene expression profiling made it obvious that currently generated kidney organoids fail to recapitulate a vascular network^5^. This is primarily caused by the lack of an established endothelial cell population and supporting cues to guide and maintain them in a complex tissue structure. Recent single-cell studies report that organoids generated by Takasato and Morizane contain only 0.1 to 0.2% endothelial cells^23^. Organoids generated with the Takasato protocol produced endogenous VEGF, likely not from the low number of endothelial cells as it is known that podocytes are the main producers of this cytokine^31,32^. Transplantation under the mouse renal capsule resulted in major improvements of glomerular and tubular structures and the formation of endothelial networks^33^. This suggests that at least part of the host vasculature connects with the organoid to join the resident endothelial progenitors. Bantounas and colleagues took a similar approach by subcutaneously implanting kidney progenitors into immunocompromised mice^24^. In accordance with van den Berg and colleagues, this engraftment showed evidence of vascularization from the host and improved maturation of glomerular structures^24^. More recently, another transplantation method was developed using the chick chorioallantoic membrane. Its soft microenvironment supported organoid growth and promoted the generation of a vascular component^16^. Although providing organoids with a host circulation promotes maturation, the implants still resemble first and second trimester human kidneys^31^. This implies vascularization is crucial but insufficient for promoting organoid maturation alone. The reliance on animal hosts to improve organoid vascularization and maturation limits both scalability and the potential for therapeutic translation. In order to counteract these shortcomings, there is an obvious need for in vitro vascularization, at least partially.

A first leap forward was achieved by using microfluidics to induce flow over the top surface of kidney organoids. By optimizing the flow conditions, the pool of endothelial progenitors was expanded and vascular networks containing lumens were generated^34^. A more challenging microfluidic approach relies in the molding of artificial capillary networks in biomaterials and hydrogels. Such channels can be coated with endothelial cells achieving vascularized networks able to supply oxygen and nutrients^35^. Hydrogels are gaining more attention for creating microfluidic channels as they offer important advantages, such as ECM-like properties, increased biocompatibility and free diffusion of small molecules^36^. Despite recent advances, microfluidic approaches still face limitations to closely mimic physiological rounded vessels and to produce sufficiently small channels^37,38^. Combining the knowledge obtained from in vivo transplantation studies with microfluidics will be needed to pave the way for the generation of vascularized organoids.

In light of these limitations, 3D bioprinting a bioengineered kidney may be a promising alternative. Printing small, rounded channels with a high spatial flexibility has been shown to be feasible for creating prevascularized tissue constructs^39,40^. But, once introduced as a very promising technique, its full potential has never been completely realized. This may be explained by the harsh conditions single cells undergo during printing, the limitations of the printable dimensions, and the current lack of printable matrices that satisfactorily reflect the biophysical and biochemical conditions.

Although microfluidics and 3D bioprinting require some additional refinement, they appear to be promising strategies to achieve tissue vascularization, which is maybe one of the biggest challenges in regenerative medicine at this moment. Once established in kidney organoids, vascularization will undoubtedly improve the structural maturation of the entire organoid, thereby opening new avenues for kidney regeneration.

### Increasing reproducibility and scaling

The human body relies on a pair of kidneys, containing each on average 900,000 to one million nephrons, to filter out uremic toxins and to maintain body fluid homeostasis^41^. Depending on age and health status, this number may vary but most of the variation is developmentally determined. The same holds true for the kidney size that can range from 134 to 146 cm^3^`^25^. Values for these parameters obtained from currently grown kidney structures are several magnitudes smaller. At present, kidney organoids generated by Takasato’s protocol are ~7 mm in diameter and contain 50–100 nephrons. There is also the probability they vary in cellular composition due to off-target populations. Until now, this patterning is uncontrolled and varies between organoid batches and donor hPSCs. This restricted scalability and poor reproducibility represent critical challenges the field needs to address before kidney organoids can meet their purpose in regenerative medicine.

In a first attempt to overcome some of these issues, Czerniecki and colleagues developed a high-throughput platform for the generation of human kidney organoids. All liquid-handling steps of the Freedman protocol were performed automatically on a robotic platform made compatible for 96- and 384-well plates. This microwell system, initially used for culturing, is also compatible for high-content imaging analysis to closely monitor organoid generation^42^. Similarly, Higgins and colleagues used a 3D bioprinter for the generation of highly reproducible organoids, suitable for high-content compound screening^43^. Two other recent approaches to upscale organoid cultures came from a recently developed bioreactor and from the creation of micro-organoids in a shaking culture platorm^44,45^. Although not every organoid protocol is suitable for suspension cultures, it offers great potential for growing organoids in large quantities and reduces culturing costs. Upscaling organoid production from limited source material needs close monitoring of mutation accumulation during cellular expansion. Together with the need to control for terminal differentiation, some major topics of discussion are left unaddressed.

## Concluding remarks

Within the past decade, significant advances were made to generate kidney organoids from hPSCs (Table 1). Explorative research for the understanding of embryogenesis and kidney morphogenesis performed on rodent models led to key publications describing the directed differentiation of hPSCs towards developing renal structures. The possibility to create a variety of renal cell types in vitro provides the opportunity for multiple applications, like drug screening, disease modeling, and renal replacement therapy. Still, currently grown kidney organoids face crucial shortcomings, as they would be improved through more maturity, functionality and reproducibility. The interface between cell biology and engineering is a particularly promising area to improve the current state of kidney organoids. Being able to control cell fate, and therefore organoid formation, by creating an optimal microenvironment composed of the required biochemical and biophysical cues will be our immediate challenge^46^. These advances will undoubtedly increase the success of next generation organoid platforms such as microfluidics and 3D bioprinting. Such emerging techniques combined with our improved knowledge of the molecular mechanisms of organ development and maturation will undoubtedly increase the suitability of kidney organoids for renal replacement therapy in regenerative medicine.

**Table 1:** Major achievements in the kidney organoid field. Overview of the most recent advances in the generation of kidney organoids.

Physiological resemblance	*Before***:** First trimester of human gestation^5^. *Now***:** Second trimester of human gestation^16^.
Vascular component	*Before***:** Endothelial markers present but no vascularization in vitro^5^. *Now***:** Development of endothelial networks and evidence of vascularization by the host upon in vivo transplantation in mice and *in ovo*^16,24,31,33^.
Ureteric bud	*Before***:** Ureteric bud markers present in vitro but no defined structures^5^. *Now***:** Development of branched ureteric bud structures interconnecting with nephrons^19^.
